# Impact of Socio-demographic Characteristics on Time in Outpatient Cardiology Clinics: A Retrospective Analysis

**DOI:** 10.1177/00469580231159491

**Published:** 2023-03-15

**Authors:** Daniel McIntyre, Simone Marschner, Aravinda Thiagalingam, David Pryce, Clara K. Chow

**Affiliations:** 1 Westmead Applied Research Centre, University of Sydney, Sydney, Australia; 2 Westmead Hospital, Sydney, Australia

**Keywords:** waiting time, inequity, cardiovascular disease, outpatient, public health

## Abstract

Inequitable access to health services influences health outcomes. Some studies have found patients of lower socio-economic status (SES) wait longer for surgery, but little data exist on access to outpatient services. This study analyzed patient-level data from outpatient public cardiology clinics and assessed whether low SES patients spend longer accessing ambulatory services. Retrospective analysis of cardiology clinic encounters across 3 public hospitals between 2014 and 2019 was undertaken. Data were linked to age, gender, Indigenous status, country of birth, language spoken at home, number of comorbidities, and postcode. A cox proportional hazards model was applied adjusting for visit type (new/follow up), clinic, and referral source. Higher hazard ratio (HR) indicates shorter clinic time. Overall, 22 367 patients were included (mean [SD] age 61.4 [15.2], 14 925 (66.7%) male). Only 7823 (35.0%) were born in Australia and 8452 (37.8%) were in the lowest SES quintile. Median total clinic time was 84 min (IQR 58-130). Visit type, clinic, and referral source were associated with clinic time (R^2^ = 0.23, 0.35, 0.20). After adjusting for these variables, older patients spent longer in clinic (HR 0.94 [0.90-0.97]), though there was no difference according to SES (HR 1.02 [0.99-1.06]) or other variables of interest. Time spent attending an outpatient clinic is substantial, amplifying an already significant time burden faced by patients with chronic health conditions. SES was not associated with longer clinic time in our analysis. Time spent in clinics could be used more productively to optimize care, improve health outcomes and patient experience.

**What do we already know about this topic?**
There is some evidence those of lower socio-economic status wait longer for elective surgery, but a paucity of data on patient time burden in the ambulatory care setting.
**How does your research contribute to the field?**
In this study of over 20 000 encounters with publicly funded outpatient cardiology clinics in Australia, 50% of patients spent at least 84 min in clinic. After controlling for clinic-related factors, there was no significant difference in time according to socio-economic status.
**What are your research’s implications toward theory, practice, or policy?**
Time spent accessing ambulatory care appears equal between socio-demographic groups, however is significant for all patients, and poorly recognized by healthcare providers. This time could be better utilized to opportunistically deliver interventions that improve population health.

## Introduction

Time spent accessing healthcare is a key measure of service quality and strain.^1,2^ Elective surgery waiting times are the focus of most analyzes,^2^ and have increased in recent years.^3^ However, patients wait in multiple settings—in the community for primary care,^4^ specialist,^5,6^ and allied health^7^ appointments, and in waiting rooms in emergency^8^ and ambulatory clinics.^9^ Compared to elective surgery, these other waiting times are poorly characterized, providing clinicians and policy makers with an incomplete view of patient time burden across healthcare systems.

This burden is greatest for patients with multiple comorbid conditions, such as cardiovascular diseases, who require increased healthcare contact.^10^ There is international evidence that elective surgery waiting times are greater for patients of lower socio-economic status (SES).^11-14^ This is particularly concerning in single payer health systems where waiting time should be allocated according to clinical acuity, rather than ability to pay. However, there are few studies on patient time burden in other settings.

Particularly, there are a lack of data on time spent accessing ambulatory care and in outpatient clinic waiting rooms. Such time may seem less significant as an absolute, but cumulates with increasing healthcare contact and has an associated opportunity cost secondary to missed work hours, estimated at 15 cents per dollar spent on healthcare.^15^ The largest reports on waiting room time are from the USA and indicate a likely time of 20 to 40 min.^9,16,17^ Some studies suggest patients from lower socio-economic backgrounds wait longer in this setting as well. An analysis of 3787 responses to the American Time Use Survey by Ray et al^18^ found time accessing outpatient care was 123 min on average and significantly longer for Black and Hispanic patients, those with less education, and the unemployed. Oostrom et al^19^ analyzed 21 million outpatient office visits in the USA, finding publicly insured (Medicaid) patients were 20% more likely than privately insured patients to wait longer than 20 min. A small 2022 analysis of 423 attendees to a public outpatient clinic in Ethiopia found those with lower educational attainment were more likely to have long waiting times than tertiary-educated participants (odds ratio 2.25 [95% CI 1.11, 4.58]).^20^ A study of 96 patients in a Nigerian outpatient department found women were more likely to experience waiting times of ≥180 min than men (31.6% vs 6.3%, respectively).^21^ While these data suggest a relationship may exist, to our knowledge, there are no studies comparing clinic time with SES in single-payer healthcare systems such as the UK, Canada, or Australia.

In this study, we present data from consecutive patients attending outpatient cardiology appointments across 3 public hospitals in Sydney, Australia between 2014 and 2019. We aim to describe the “clinic time” (difference between time arrived and time departed) and assess whether this is impacted by socio-demographic characteristics including SES, age, gender, number of comorbidities, country of birth, and language spoken at home.

## Methods

### Setting and Study Population

We examined a consecutive patient-level data set of all public outpatient cardiology encounters across 3 hospitals within Western Sydney Local Health District (WSLHD) between July 2014 and December 2019. Clinics are consultant-led and staffed by junior doctors, training cardiologists, and nursing staff. Patients are referred by general practitioners, emergency departments, or other doctors and generally do not pay to access these clinics. WSLHD comprises 5 hospitals, 7 community health centers, and serves 946 000 residents in the western suburbs of Sydney.^22^ The population is diverse with 46.8% of residents born overseas and 50.3% speaking a language other than English. WSLHD also houses the largest Aboriginal and Torres Strait Islander population in Australia (approximately 13 000 persons).^22^

### Inclusion and Exclusion Criteria

All adult (>18) patients who accessed outpatient cardiology services in-person across WSLHD between July 2014 and December 2019 were included in the analysis.

Patients were excluded if their clinic time was not assessed. This was defined if clinic time data were missing, equal to 0, or if all patients within a clinic were allocated to a pre-specified time (eg, 30, 45, or 60 min). Extreme values were excluded with cut-offs of ≤20 min (the presumed time of a consultation only), or ≥240 min (the entire duration of a morning or afternoon clinic session) as these times were likely due to data entry error or unreliable clerical processes. Audio and inpatient consultations were excluded.

### Data Collection, Handling, and Definitions

The data were cleaned, de-identified and processed by the Business Analytics Service (BAS) at Westmead Hospital and passed to the Westmead Applied Research Center, University of Sydney, via a secure server. The data contained patient-level variables on age, gender, Indigenous status, country of birth, language spoken at home, number of comorbidities, and postcode. Data on country of birth, Indigenous status, and language spoken is obtained from all patients via self- report on presentation to hospital. Patient postcode was correlated with the 2016 socio-economic indexes for areas (SEIFA) Index of relative socio-economic disadvantage (IRSD) score. This score is derived from 2016 Australian census data and summarizes variables that indicate relative disadvantage. The lower the score, the higher proportion of disadvantaged people reside within the postcode of interest.^23^ IRSD deciles were applied to each patient for the final analysis. In addition, the data contained appointment-level information on time of day, visit type (new or follow up), referrer (emergency department or other), clinic type (arbitrarily categorized A-R for consultant and hospital anonymity), arrived time, and departed time.

Total clinic time was calculated by measuring the difference between time arrived and time departed. This is a convenience measure taken by administration staff as part of the normal clinic workflow.

### Statistical Analysis

Statistical analysis was undertaken using R statistical software (V3.6.1). All variables of interest were first interrogated visually to assess for normality of distribution. Means were calculated for normally distributed continuous variables, and medians for non-normal continuous variables. Categorical variables were presented as frequencies and percentages.

Initially, the proportion of patients waiting longer than the median clinic time in different demographic groups (Age ≥75 vs <75, IRSD ≤5 vs >5, ≥4 comorbidities vs <4, female vs male, Indigenous vs non-Indigenous, born in Australia vs born Overseas, and English vs other language spoken at home) was compared with a chi-squared test. A univariate unadjusted linear regression was then conducted on the above patient characteristics and clinic process measures (clinic, visit type (new/follow up), referrer, appointment year, and time of day) to determine variables associated with increased clinic time.

A cox proportional hazard model was then applied to identify patient-level predictors of increased time in clinic. The model outcome was the time the patient left clinic. A higher hazard ratio (HR) described greater chance of leaving clinic earlier and hence shorter total time in clinic. This analytic approach was selected due to the non-normal distribution of the time data and is similar to cox proportional hazard models applied to assess time to wound healing, where a higher HR corresponds to a better outcome.^24^ Multivariate models controlled for clinic, visit type, referral source, and the above demographic characteristics. Results of these models are presented as HRs with 95% confidence interval (CIs). Further analysis was conducted to identify interactions between patient and clinic-level variables of interest. Finally, within-hospital and within-clinic (shorter wait versus longer wait) analysis was conducted to determine whether discrepancies could be accounted for by between-hospital and clinic differences.

## Results

**Figure 1.**
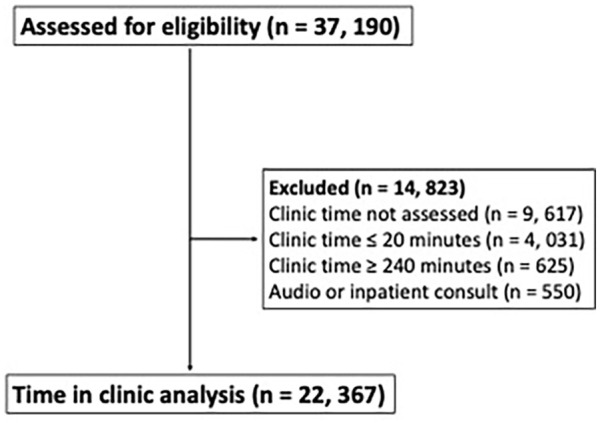
Inclusion/exclusion of patients for the final analysis.

Of 37 456 patients assessed for eligibility, 14 823 were excluded and 22 367 were included in the final analysis (Figure 1). Of these, 14 925 (65.9%) were male and the mean age was 61.4 (SD 15.2) years. Only 7823 (35.0%) were born in Australia, and 8452 (37.8%) were in the lowest IRSD decile, indicating they resided in a postcode with a greater proportion of disadvantaged residents than 90% of postcodes in Australia. A significant proportion of patients had >4 comorbidities (40.4%). Cardiac risk factors and comorbid cardiac conditions were also relatively common (Table 1).

**Table 1.** Population Characteristics.

Parameter	Patients attending outpatient cardiac services Mean (SD) NN (%) Total = 22 367
Demographic	
Age, years	61.4 [15.2]
Age ≥ 75	4075 (18.2%)
Male	14 925 (66.7%)
Born in Australia	7823 (35.0%)
Primary language English	15 946 (71.3%)
Aboriginal or Torres Strait Islander	319 (1.4%)
IRSD quintile	
First	8452 (37.8%)
Second	3264 (14.6%)
Third	2492 (11.1%)
Fourth	4607 (20.6%)
Fifth	3550 (15.9%)
No fixed address	2 (0.01%)
Hospital	
A	13 405 (59.9%)
B	1936 (8.7%)
C	7026 (31.4%)
Appointment type	
New	7084 (31.7%)
Follow-up	15 283 (68.3%)
Referrer—new appointments	
Medical/surgical specialist	2155 (9.6%)
General practitioner	3979 (17.8%)
Emergency department	5185 (23.2%)
Other agency	353 (1.6%)
No referral source documented	10 695 (47.8%)
Appointment details	
Morning	16 253 (72.7%)
Afternoon	6114 (27.3%)
Appointment time (median [IQR] minutes)	84 [58-130]
Current comorbidities	
0	2222 (9.9%)
1-4	10 994 (49.2%)
>4	9151 (40.9%)
Median [IQR] number of comorbidities	4 (2-7)
Cardiac risk factors	
Hypertension	10 856 (48.5%)
Diabetes mellitus	6895 (30.8%)
Smoking	2670 (11.9%)
Obesity	2577 (11.5%)
Alcohol misuse disorder	237 (1.1%)
Cardiac comorbidities	
Heart failure	1917 (8.6%)
Ischemic heart disease	4508 (20.2%)
Atrial fibrillation/atrial flutter	1160 (5.2%)
Any cardiac comorbidity	6455 (28.9%)
Non-cardiac comorbidities	
Chronic obstructive pulmonary disease	2910 (13.0%)
Chronic kidney disease	1954 (8.7%)
Obstructive sleep apnea	176 (0.8%)
Cancer; malignant	350 (1.6%)

*Note*. IRSD = Index of relative socio-economic disadvantage (lower indicates more disadvantage); IQR = Interquartile range.

### Time Spent in Clinic

**Figure 2.**
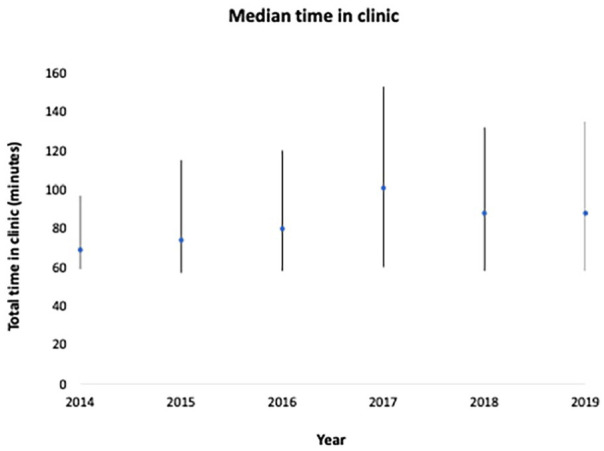
Median [interquartile range] time in clinic according to appointment year.

The median total time in clinic was 84 min (interquartile range 58-130). The distribution was flat across the years of observation, ranging from 69 min in 2014 to 101 min in 2017 (Figure 2).

### Process Measures as Predictors of Longer Time in Clinic

Clinic process measures were analyzed for their association with clinic time. New patients and those referred from the emergency department were the most likely to spend longer in clinic (median 120 and 125 min, respectively, Figure 2). There was significant variance between clinics (Table 2). Linear regression demonstrated low to moderate association between all process measures and clinic time besides year of appointment and time of day (Table 2). Visit type, clinic, and referral source account for 23.0%, 35.0%, and 20.0% of the variance (R^2^) in clinic time, respectively.

**Table 2.** Association of Cardiology Clinic Process Measures With Waiting Time.

	N (%) Total = 22 367	Median (IQR) clinic time	Overall R^2^	*F*-statistic	*F*-statistic *P*-value
Visit type			0.23	749	<0.01
Follow-up	15 283 (68.3)	69 (54-110)			
New	7084 (31.7)	120 (86-159)			
Clinic			0.35	213	<0.01
A	429 (1.9)	94 (65-129)			
B	74 (0.3)	83 (61-120)			
C	17 (0.1)	135 (80-158)			
D	139 (0.6)	97 (78-124)			
E	6777 (30.3)	58 (50-68)			
F	249 (1.1)	40 (29-58)			
G	128 (0.6)	45 (30-51)			
H	12 (0.1)	30 (30-35)			
I	352 (1.6)	42 (30-59)			
J	343 (1.5)	148 (106-186)			
K	1408 (6.3)	72 (53-105)			
L	1296 (5.8)	90 (60-137)			
M	6946 (31.1)	127 (92-169)			
N	25 (0.1)	80 (63-112)			
O	11 (0.0)	55 (49-70)			
P	1104 (4.9)	101 (74-136)			
Q	1373 (6.1)	107 (82-140)			
R	1684 (7.5)	102 (76-133)			
Referral source			0.20	213	<0.01
Emergency department	5185 (23.2)	125 (91-168)			
Other	17 182 (76.8)	72 (55-116)			
Year			0.03	1.68	0.2
2014	1380 (6.2)	69 (59-97)			
2015	3011 (13.5)	74 (57-115)			
2016	3945 (17.6)	80 (58-120)			
2017	4568 (20.4)	101 (60-153)			
2018	4680 (20.9)	88 (58-132)			
2019	4783(21.4)	88 (58-135)			
Time of day			0.004	271	<0.01
Morning	16 253 (72.7)	76 (55-130)			
Afternoon	6114 (27.3)	98 (72-130)			

*Note*. IQR = interquartile range.

### Patient-Level Predictors of Time in Clinic

**Figure 3.**
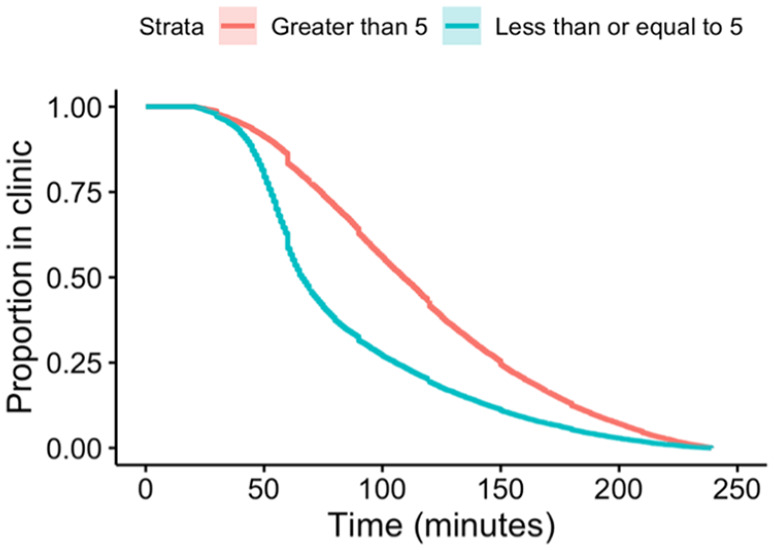
Survival plot: Socio economic status (IRSD ≤5 vs >5) and time leaving clinic. Unadjusted model.

All patient-level variables were assessed for their correlation with clinic time in a multivariate cox proportional hazards model controlling for clinic, referral source and visit type. In the unadjusted model, low (IRSD ≤ 5th decile) SES patients spent less time in clinic than those of high (IRSD > 5th decile) SES (median 66 min vs 109 min, Figure 3). After adjustment, this was no longer significant (HR 1.02 [0.99-1.06]). Those older than 75 were less likely to leave the clinic (HR 0.94 [0.90-0.97). The relationship between all other sociodemographic characteristics did not reach significance after adjustment (Table 3).

**Table 3.** Cox Proportional Hazard Model of Time Spent in Clinic and Participant Characteristics.

	N (%) Total = 22 367	Median (IQR) clinic time	Unadjusted HR [95% CI]	Adjusted* HR [95% CI]
Age				
≤ 75 (ref)	18 292 (81.8%)	86 (59-132)	1.13 [1.09-1.17]	0.94 [0.91-0.97]
> 75	4075 (18.2%)	77 (57-120)		
IRSD				
>5th decile (ref)	12 266 (54.8%)	109 (74-150)	1.73 [1.68-1.78]	1.02 [0.99-1.05]
≤5th decile	10 101 (45.2%)	66 (53-106)		
Number of comorbidities				
≤4 (ref)	13 216 (59.1%)	89 (60-136)	1.16 [1.13-1.19]	0.99 [0.97-1.02]
>4	9151 (40.9%)	79 (57-122)		
Gender				
Female (ref)	7442 (33.3%)	97 (62-141)	1.25 [1.21-1.28]	1.01 [0.98-1.04]
Male	14 925 (66.7%)	77 (56 -124)		
Indigenous status				
Non-indigenous (ref)	22 048 (98.6%)	84 (58-130)	0.95 [0.85-1.06]	1.08 [0.96-1.21]
Indigenous	319 (1.4%)	90 (64-132)		
Country of birth				
Australia (ref)	7823 (35.0%)	89 (60-134)	1.09 [1.06-1.12]	1.03 [0.99-1.06]
Other	14 544 (65.0%)	81 (57-127)		
Language spoken at home				
English (ref)	15 946 (71.3%)	88 (60-133)	1.14 [1.11-1.17]	0.99 [0.96-1.02]
Other	6421 (28.7%)	76 (55-122)		
Primary diagnosis				
Non-cardiac (ref)	15 745 (70.4%)	89 (60-135)	1.18 [1.15-1.21]	1.00 [0.97-1.03]
Cardiac	6622 (29.6%)	76 (55-120)		

*Note*. IRSD = index of relative socio-economic disadvantage (lower indicates more disadvantage); HR = hazard ratio; IQR = interquartile range.

* Adjusted for clinic, visit type, referral source, and demographic characteristics.

### Interaction Analysis of Demographic, Process Measures, and Socio-Economic Status

Further analysis was performed assessing the interaction between SES, patient characteristics and clinic process measures. Those of lower SES spent less time in clinic irrespective of their age, gender, number of comorbidities, country of birth or language spoken at home. However, after adjustment for visit type, clinic, and referral source, there was no interaction between SES and any of the identified demographic variables (Supplemental Table 1). Patients of lower SES were more likely to attend follow-up appointments (77.2% vs 57.6%), clinics with short clinic time (66.8% vs 21.1%) and be referred from sources other than the emergency department, compared to patients of higher SES (Supplemental Table 1).

### Clinic and Hospital Sub Analysis

To assess for discrimination within hospitals and clinics, the association between socio-economic status and time in clinic was analyzed in a further cox proportional hazards model adjusted for clinic, referral source and visit type. Those of lower SES spent slightly less time in clinics in hospital C (57 min vs 60 min, HR 1.24 [1.13-1.37]), though there were no differences within other hospitals. Within short wait clinics, lower SES spent less time in clinic (59 min vs 71 min, HR 1.10 [1.05-1.17]). There was no difference according to SES in longer wait clinics (Supplemental Table 2).

## Discussion

This analysis of over 20 000 consecutive outpatient cardiology clinic encounters aimed to determine whether those of low SES were more likely to spend longer in clinic. After adjusting for visit type, clinic, and referral source, there was no difference in clinic time according to SES. Overall, 75% of patients spent at least 1hour in clinic. One quarter spent more than 2 hours. Potential implications of these findings include consideration of a more productive use of this time in ambulatory clinics, such as implementing interventions during this time that can improve health literacy and may improve health outcomes and satisfaction with health services.^25,26^

The interaction between SES and time to accessing health services has been debated for over 20 years. Most data are derived from elective surgery waiting lists,^13,27^ and there is some evidence discrimination is reversing as new policies are introduced. Cooper et al^28^ analyzed elective surgery wait lists in 1997 to 2000, 2001 to 2004, and 2005 to 2007, finding the effect of SES on waiting time reduced over the period of observation and reversed for knee replacement and cataract repair in 2005 to 2007, such that the most deprived fifth waited less than the least deprived fifth. There are less studies of the Australian system, but most reports suggest discrimination. Johar et al^29^ studied 90 162 patients in New South Wales public hospitals, finding that more advantaged patients waited less for elective surgery at all quintiles of waiting time. Data from developing countries is also suggestive of discrimination in this setting. A 2017 analysis of 219 surgeries within an Indian teaching hospital found those living below the poverty line had threefold higher waiting times than those above the poverty line.^30^ However, data are very limited within developing countries, largely due to a lack of systematic reporting. For example, a recent international collaboration for systematic reporting of waiting times is limited to organization for economic co-operation and development (OECD) countries, which are almost exclusively high-income.^31^

The finding of no relation to SES for patients accessing public clinics in our study is reassuring and may be explained by several reasons. There are likely fewer opportunities for preferential treatment within waiting rooms (where patients are seen in the order they arrive) than elective surgery (where waiting time is determined by clinician priority allocation), which may explain the lack of association between SES and clinic time in our study.

The Australian system is private-public, where patients with insurance that anticipate a long wait time can opt-in for private hospital care. There is evidence this preferential service selection model explains elective surgery waiting time inequity in Australia,^32^ though more studies of waiting room time are needed. Many hospitals in Australia run large public outpatient services where patients generally do not pay out-of-pocket for services, which are the services analyzed here. However, higher SES patients are more likely to access privately billed clinics in the community and findings here may have limited applicability to these care settings. They do however suggest that the lack of relation to SES of time spent in public clinics found here may be because of the absence of per-patient payment and of classification based on public/private status.

Patients with cardiovascular disease are more likely to be older, Indigenous, of lower SES, live in rural areas and have comorbidities than the general population.^33^ Analysis of time in cardiology clinics provides an opportunity to assess for poorer outcomes among these patient populations. In our study, we found patients older than 75 were more likely to spend longer in cardiology clinics. This may be due to these patients having more complex care needs requiring a longer consultation with additional time to see other health professional, for example, nurses, allied health workers, social workers. Older patients may also be more likely to arrive early to clinic appointments, increasing the overall appointment time. Faiz and Kristoffersen^34^ collected data from 1353 outpatient neurology clinic appointments and found older patients were less likely to arrive late than younger patients (OR 0.74 [0.63-0.88]).

In our study, lower SES patients were more likely to attend follow-up appointments and clinics with shorter waits overall, both strong predictors of reduced total clinic time. Sub-analysis of these clinics found lower SES patients spent less time after adjusting for process measures. Importantly, our analysis did not delineate between consultation and waiting room time. It is possible that lower SES patients had shorter consult times, which was the primary driver for a shorter total clinic time. This is supported by an analysis of 70 758 GP consultations in Australia in 2001 to 2002, which found older patients of higher SES had longer consultation times.^35^ A 2020 qualitative analysis of 36 head and neck cancer appointments found lower SES patients were more passive in their care, engaging in less agenda setting and information seeking, potentially explaining shorter consultation times within this group.^36^ Further studies are needed to better define patient time burden while waiting, an indicator of poor care, from time spent with clinicians, likely an indicator of quality care.

The implications of “in-clinic” waiting times are different to those for elective surgery, specialist and primary care visits, where longer waiting time has been associated with poorer clinical outcomes.^37-39^ Increased time in ambulatory care has been linked to reduced care satisfaction,^40^ however the consequences are primarily economic – the opportunity cost of accessing healthcare. Increasing workforce casualization, where employees do not have access to sick leave, further compounds the economic cost of increased clinic time.^41^ These implications are greater for patients that require more contact with healthcare services.

### Addressing Patient Waiting Time—What Approaches Are Needed?

Several methods have been trialed to reduce the time patients spend accessing healthcare. In the emergency department, the introduction of 4-hour targets in the UK, Australia and other countries has seen significant reductions in waiting times.^42^ However, there may be diminishing returns from further reductions. Sullivan et al^43^ present an analysis of 12.5 million emergency department episodes of care, finding compliance with waiting time targets reduced in-hospital mortality. However as compliance increased past a critical point of 83%, the relationship was lost. Countries that lack a benchmark likely have even longer waiting times. A 2006 analysis of 675 patients at a public hospital in Barbados revealed a median 377 min length of stay, over 2 hours longer than targets in Australia and the UK.^44^ Despite some small studies in China,^45^ Singapore,^46^ and Korea,^47^ there is a paucity of research about interventions to address in-clinic waiting time. To our knowledge, there are no examples of such interventions within cardiology outpatient clinics.

Irrespective of between-group differences, this study underscores that time spent accessing healthcare is significant. This time could be better utilized to deliver health interventions that convert this from wasted to productive time. There is some literature suggesting waiting room interventions can improve patient knowledge, but a paucity of robustly designed studies to assess the efficacy of waiting room interventions on clinical outcomes.^48,49^ Though a focus on health outcomes is desirable, waiting room interventions could also target process outcomes such as patient satisfaction with care, total time in clinic or consultation time. Integrated delivery of tech-enabled interventions that begin in the waiting room, continue through the consultation and into the post-consultation period could contribute to a new paradigm of healthcare that values patient time whilst also increasing provider efficiency.^50^

There are several strengths and weaknesses to this study. We considered a consecutive sample of patients attending a single specialty within one local health district. This limited between-hospital and specialty heterogeneity, however provided limited view on waiting times in rural locations, other cities and specialties. Data were collected over 5 years, providing insight into longitudinal waiting time trends within our sample and were convenience based and likely less prone to bias than data collected by self-report or specifically measured for the monitoring of waiting time. The convenience nature of these data also limits generalizability. Approximately 40% of encounters where data were incomplete or unreliable were excluded to minimize impact on findings (Figure 1). We did not have differential data on time spent with clinicians versus in waiting rooms and could not identify patients that left clinic without being seen by a doctor. We were unable to characterize the urgency of each patient’s clinic visit and cannot rule out an effect due to preferential treatment of higher acuity patients. A sample size calculation was also not performed in this study. All available data in the sample were analyzed. Finally, data were at the level of the encounter, not the patient. It is possible there are duplicate patients who attended clinics multiple times within the data set.

## Conclusions

Accessing healthcare presents a significant time burden for patients at all levels of the health system. In this analysis of 22 367 patients attending publicly funded outpatient cardiology clinic appointments over 6 years, older patients spent longer in clinic, but no difference for low SES or other demographically disadvantaged patients was identified. This is reassuring, however does not exclude the possibility of disparities. Further studies that are prospective and diverse in geographical, health service funding, and economic advantage at a country level are required. Ongoing monitoring of the health system with respect to performance and inequities is also important. Consideration should be given to the opportunistic delivery of interventions during this time to improve health engagement and outcomes.

## Supplemental Material

sj-docx-1-inq-10.1177_00469580231159491 – Supplemental material for Impact of Socio-demographic Characteristics on Time in Outpatient Cardiology Clinics: A Retrospective Analysis Click here for additional data file. Supplemental material, sj-docx-1-inq-10.1177_00469580231159491 for Impact of Socio-demographic Characteristics on Time in Outpatient Cardiology Clinics: A Retrospective Analysis by Daniel McIntyre, Simone Marschner, Aravinda Thiagalingam, David Pryce and Clara K. Chow in INQUIRY: The Journal of Health Care Organization, Provision, and Financing

sj-docx-2-inq-10.1177_00469580231159491 – Supplemental material for Impact of Socio-demographic Characteristics on Time in Outpatient Cardiology Clinics: A Retrospective Analysis Click here for additional data file. Supplemental material, sj-docx-2-inq-10.1177_00469580231159491 for Impact of Socio-demographic Characteristics on Time in Outpatient Cardiology Clinics: A Retrospective Analysis by Daniel McIntyre, Simone Marschner, Aravinda Thiagalingam, David Pryce and Clara K. Chow in INQUIRY: The Journal of Health Care Organization, Provision, and Financing
