# Supplementary material for: Impact of Socio-demographic Characteristics on Time in Outpatient Cardiology Clinics: A Retrospective Analysis
Source: Inquiry. 2023 Mar 15;60:00469580231159491. doi: 10.1177/00469580231159491 (PMC10021097; doi:10.1177/00469580231159491)
Supplement: sj-docx-1-inq-10.1177_00469580231159491 – Supplemental material for Impact of Socio-demographic Characteristics on Time in Outpatient Cardiology Clinics: A Retrospective Analysis [file sj-docx-1-inq-10.1177_00469580231159491.docx]

|  | N (%)  Total = 22,367 | Median (IQR) clinic time | HR (adjusted*) | N (%) | Median (IQR)  clinic time | HR (adjusted*) |
| --- | --- | --- | --- | --- | --- | --- |
|  | IRSD ≤ 5 |  |  | IRSD > 5 |  |  |
| **Age** | | | | | | |
| ≤ 75 | 9,934 (81.0%) | 67 (53 – 110) | 1.05 [1.00 – 1.10] | 8,358 (82.7%) | 110 (74 – 150) | 1.03 [0.98 – 1.08] |
| > 75 | 2,332 (19.0%) | 63 (53 – 90) | REF | 1,743 (17.3%) | 107 (72 – 148) | 0.94 [0.88 – 1.01] |
| **Gender** | | | | | | |
| Female | 3,604 (29.4%) | 80 (56 – 123) | 0.97 [0.93 – 1.01] | 3,838 (38.0%) | 113 (78 – 153) | 0.97 [0.93 – 1.02] |
| Male | 8,662 (70.6%) | 63 (52 – 96) | REF | 6,263 (62.0%) | 107 (72 – 149) | 0.96 [0.93 – 1.00] |
| **Comorbidities** | | | | | | |
| ≤4 | 6,872 (56.0%) | 68 (54 – 110) | 1.03 [0.99 – 1.07] | 6,344 (62.8%) | 112 (76 – 153) | 0.99 [0.95 – 1.03] |
| > 4 | 5,394 (44.0%) | 65 (52 – 100) | REF | 3,757 (37.2%) | 104 (70 – 144) | 0.99 [0.95 – 1.04] |
| **Country of birth** | | | | | | |
| Australia | 3,921 (32.0%) | 70 (55 – 112) | 0.95 [0.92 – 0.99] | 3,902 (38.6%) | 108 (75 – 150) | 0.96 [0.92 – 1.00] |
| Other | 8,345 (68.0%) | 65 (52 - 103) | REF | 6,199 (61.4%) | 109 (74 – 150) | 0.96 [0.92 – 0.99] |
| **Language spoken at home** | | | | | | |
| English | 8,159 (66.5%) | 68 (54 – 110) | 0.98 [0.94 – 1.02] | 7,787 (77.1%) | 109 (74 – 150) | 0.97 [0.93 – 1.01] |
| Other | 4,107 (33.5%) | 64 (52 – 97) | REF | 2,314 (22.9%) | 109 (74 - 150) | 0.93 [0.88 – 0.99] |
| **Indigenous status** | | | | | | |
| Non-Indigenous | 12,059 (98.3%) | 66 (53 – 105) | 0.99 [0.86 – 1.14] | 9,989 (98.9%) | 109 (74 – 150) | 0.97 [0.84 – 1.11] |
| Indigenous | 207 (1.7%) | 90 (64 – 130) | REF | 112 (1.1%) | 90 (64 – 132) | 1.17 [0.93 – 1.18] |
| **Visit type** | | | | | | |
| Follow up | 9,486 (77.2%) | 61 (51 – 82) | 2.05 [1.96 – 2.14] | 5,815 (57.6%) | 98 (66 – 138) | 1.68 [1.60 – 1.76] |
| New | 2,798 (22.8%) | 116 (81 – 151) | REF | 4,286 (42.4%) | 121 (88 – 164) | 1.35 [1.28 – 1.42] |
| **Clinic** | | | | | | |
| Short wait | 8,202 (66.8%) | 59 [49 – 71] | 3.03 [2.87 – 3.19] | 2,130 (21.1%) | 71 [55 – 115] | 1.76 [1.66 – 1.88] |
| Long wait | 4,064 (33.1%) | 116 [83 – 158] | REF | 7,971 (78.9%) | 117 [ 85 – 158] | 1.00 [0.96 – 1.04] |
| **Referral source** | | | | | | |
| Other | 10,712 (87.3%) | 62 (51 – 90) | 1.01 [0.95– 1.08] | 6,470 (64.1%) | 98 (65 – 137) | 0.98 [0.92 – 1.04] |
| Emergency | 1,554 (12.7%) | 124 (90 – 168) | REF | 3,631 (35.9%) | 126 (92 – 168) | 0.99 [0.93 – 1.05] |

*Supplementary table 1: Interaction analysis between in-clinic time and socio-economic status, demographic and process variables. IRSD = Index of relative socio-economic disadvantage (lower indicates more disadvantage), HR = hazard ratio, IQR = Interquartile range, *Adjusted for clinic, visit type, referral source, and demographic characteristics*
