# Supplementary material for: Impact of Socio-demographic Characteristics on Time in Outpatient Cardiology Clinics: A Retrospective Analysis
Source: Inquiry. 2023 Mar 15;60:00469580231159491. doi: 10.1177/00469580231159491 (PMC10021097; doi:10.1177/00469580231159491)
Supplement: sj-docx-2-inq-10.1177_00469580231159491 – Supplemental material for Impact of Socio-demographic Characteristics on Time in Outpatient Cardiology Clinics: A Retrospective Analysis [file sj-docx-2-inq-10.1177_00469580231159491.docx]

|  | N (%) | Median (IQR) clinic time | N (%) | Median (IQR) clinic time | HR (adjusted*) |
| --- | --- | --- | --- | --- | --- |
| **Hospital** | IRSD > 5 (REF) |  | IRSD ≤ 5 |  |  |
| A | 8,883 (66.2%) | 116 (83 – 155) | 4,522 (33.8%) | 114 (81 – 156) | 1.00 [0.96 - 1.03] |
| B | 770 (39.8%) | 65 (45- 97) | 1166 (40.2%) | 62 (44 – 90) | 1.06 [0.97 – 1.17] |
| C | 448 (6.4%) | 60 (51 – 75) | 6,578 (93.6%) | 57 (49 – 67) | 1.26 [1.14 – 1.39] |
| **Clinic** | | | | | |
| Short wait | 2,130 (20.6%) | 71 (55 – 115) | 8,202 (79.4%) | 59 (49 – 71) | 1.11 [1.05 – 1.17] |
| Long wait | 7,971 (66.2%) | 117 (85 – 158) | 4,064 (33.8%) | 116 (83 – 158) | 0.99 [0.96 – 1.03] |

*Supplementary table 2: Within hospital and within clinic analysis of socio-economic status and in-clinic time. IRSD = Index of relative socio-economic disadvantage, IQR = Interquartile range, HR = hazard ratio, *Adjusted for clinic, visit type, referral source, and demographic characteristics*
